# The impact of cholesteryl ester transfer protein on the progression of cutaneous leishmaniasis

**DOI:** 10.3389/fimmu.2024.1389551

**Published:** 2024-06-20

**Authors:** Francisca Elda Batista-Dantas, Christiane Yumi Ozaki, Kelly Gomes Santana, Valéria Sutti Nunes, Bernardina Amorin Uscata, Cinthia Siess-Portugal, Luiza Campos Reis, Edite H. Yamashiro-Kanashiro, Wagner Luiz Tafuri, Amaro Nunes Duarte-Neto, Mirian Nacagami Sotto, Hiro Goto, Patrícia Miralda Cazita

**Affiliations:** ^1^ Laboratorio de Lipides (LIM10), Hospital das Clinicas HCFMUSP, Faculdade de Medicina, Universidade de Sao Paulo, Sao Paulo, SP, Brazil; ^2^ Instituto de Medicina Tropical, Faculdade de Medicina, Universidade de São Paulo, São Paulo, São Paulo, Brazil; ^3^ Facultad de Medicina, Universidad Nacional Toribio Rodriguez de Mendoza de Amazonas, Chachapoyas, Peru; ^4^ Departamento de Patologia Geral, Instituto de Ciências Biológicas, Universidade Federal de Minas Gerais, Belo Horizonte, MG, Brazil; ^5^ Departamento de Patologia, Faculdade de Medicina, Universidade de São Paulo, São Paulo, Brazil; ^6^ Departamento de Medicina Preventiva, Faculdade de Medicina, Universidade de São Paulo, São Paulo, Brazil

**Keywords:** cholesteryl ester transfer protein, cutaneous leishmaniasis, *Leishmania L. amazonensis*, mice, infection, lipoprotein, macrophage, arginase

## Abstract

**Introduction:**

Pathogenesis of cutaneous leishmaniases involves parasite growth, persistent inflammation, and likely participation of lipoproteins (LP). The cholesteryl ester transfer protein (CETP), involved in LP remodeling, has been shown to participate in the inflammatory response and the evolution of infectious conditions.

**Methods:**

We evaluated the impact of the presence of CETP on infection by *Leishmania (L.) amazonensis* in an experimental model of cutaneous leishmaniasis using C57BL6/J mice transgenic for human CETP (CETP), having as control their littermates that do not express the protein, wild-type (WT) mice. The progression of the lesion after infection in the footpad was monitored for 12 weeks. Two groups of animals were formed to collect the plantar pad in the 4th and 12th week post-infection.

**Results:**

The lesion increased from the 3rd week onwards, in both groups, with a gradual decrease from the 10th week onwards in the CETP group compared to the WT group, showing a reduction in parasitism and an improvement in the healing process, a reduction in CD68+ cells, and an increase in CD163+ and CD206, characterizing a population of M2 macrophages. A reduction in ARG1+ cells and an increase in INOS+ cells were observed. During infection, the LP profile showed an increase in triglycerides in the VLDL fraction in the CETP group at 12 weeks. Gene expression revealed a decrease in the CD36 receptor in the CETP group at 12 weeks, correlating with healing and parasite reduction. *In vitro*, macrophages derived from bone marrow cells from CETP mice showed lower parasite load at 48 h and, a reduction in arginase activity at 4 h accompanied by increased NO production at 4 and 24 h compared to WT macrophages, corroborating the in vivo findings.

**Discussion:**

The data indicate that the presence of CETP plays an important role in resolving *Leishmania (L.) amazonensis* infection, reducing parasitism, and modulating the inflammatory response in controlling infection and tissue repair.

## Introduction

Leishmaniases are diseases caused by protozoa of the genus *Leishmania*. Prevalent in tropical and subtropical regions of the world, more than one billion people are at risk of infection, and it is estimated that 30,000 new cases of visceral leishmaniasis (VL) and more than one million new cases of cutaneous leishmaniasis (CL) occur annually (1). In Brazil, 12,953 cases of CL and 1,684 cases of VL were reported in 2022 (2).

In leishmaniases, studies focusing on adaptive immune response are predominant (3), but it is known that elements outside the adaptive immune system play an important role in the development of infection. In the development of *Leishmania (Leishmania) infantum* infection, which causes VL, the vast majority of infected people do not develop the active disease, and it is known that factors other than the adaptive immune response have an important role in the development of the infection (4), and lipids arose among these factors. Some studies further show the importance of cholesterol and its fractions in VL, both in the internalization of *Leishmania* by the host cell, in the energetic metabolism of the parasite, and in the susceptibility to the disease (5, 6).

Besides, we have seen the association of lipoprotein level alterations and disease development in a human study. We have shown the association of plasma lipoprotein levels and polymorphisms in the gene encoding lipoprotein lipase with the susceptibility to the development of active VL in a case-control study with individuals from the endemic area for VL (7).

Despite advances in the studies on lipid participation in parasite metabolism and the interaction of *Leishmania* and host cells in VL, neither studies of alterations of serum lipoprotein levels nor changes in the metabolic pathway of lipoproteins influencing infection are known in cutaneous leishmaniasis. However, although different from the *Leishmania* species causing CL, the biological processes likely share similarities to those of viscerotropic *Leishmania* species and may need lipids as an energy source (8). In a study on the lipidome of *L. infantum*- or *L. amazonensis*-infected J774 mouse cells and promastigotes, it was seen the importance of a higher content of phosphatidylethanolamine plasmalogens in *L. infantum* compared with *L. amazonensis* promastigotes suggesting the importance of lipid components in the development of different clinical features of the disease (8). Studies focusing on lipid metabolism in intracellular amastigotes and studies analyzing disease development in human or experimental CL focusing on lipid metabolism are so far unknown.

To define the focus of the study, we considered the pathogenic mechanism of cutaneous leishmaniasis in humans mostly dependent on an inflammatory process initiated with activation of TH-1 type immune response, essential for parasite control, but causes tissue damage with its persistence (9).

Among the different targets of lipid metabolism, cholesteryl ester transfer protein (CETP) was chosen, considering its role in lipid metabolism and inflammatory process. CETP activity reduces plasma concentrations of high-density lipoprotein (HDL) cholesterol, a correlate of an increased risk of atherosclerotic events (10). However, in addition to being an important component of reverse cholesterol transport, recent discoveries have shown its importance in playing an anti-inflammatory role, influencing the development of infections in experimental studies of endotoxemia and sepsis (11–13). Further, the expression of CETP in macrophages promotes an intracellular antioxidant state, reduces the accumulation of free cholesterol and phagocytosis, and attenuates pro-inflammatory gene expression (14, 15).

In addition, in our study on pulmonary emphysema, we observed that the presence of CETP alters the polarization of macrophage (16).

In the present study, we investigated the impact of CETP expression on infection development and the inflammatory response in murine CL. Transgenic mice for human CETP and their wild-type C57BL6/J controls (WT) were infected by *Leishmania (L.) amazonensis.* Parasitism was evaluated, and it was correlated with inflammatory markers. We also observed alterations in lipoprotein levels and the expression of genes related to lipid transport, suggesting the implication of lipids in the development of CL.

Modulation of infection in the presence and absence of CETP was also assessed in the *in vitro* culture of murine macrophages infected with *L. (L.) amazonensis* promastigotes. The parasitism, arginase activity, and nitric oxide were evaluated.

Our data highlight the crucial role of CETP in *Leishmania (L.) amazonensis* infection development, likely influencing the immune response and promoting tissue repair.

## Materials and methods

### Animal model

All the experimental protocols were performed in accordance with the National Council for Animal Experimentation Control (CONCEA) and were approved by the Institutional Animal Care and Use Committee (CEUA) of Faculdade de Medicina da Universidade de São Paulo (FMUSP) protocol number (CEUA- FMUSP #1683/2021). CETP transgenic (Tg) mice (line 5203, C57BL6/J background) (17) were prepared using a CETP minigene linked to the natural flanking sequences of the human CETP gene by using a transgene containing 3.2 kb of upstream and 2.0 kb of downstream flanking sequence. The animal matrices were kindly provided by Prof. Helena C. F. Oliveira (University of Campinas, São Paulo, Brazil), and a breeding colony was established at the animal facility of our institution. Heterozygous human CETP-Tg mice were crossbred to generate CETP-Tg and non-Tg wild-type (WT) littermates. CETP genotyping was performed by polymerase-chain-reaction (PCR) using tail tip DNA samples according to the Jackson Laboratory modified protocol (16). The animals were housed in a conventional animal facility at 22 ± 2°C under a 12 h light/dark cycle with free access to a pelleted regular chow diet (Nuvital Quimtia, CR1, Colombo, PR, Brazil) and filtered tap water. Blood samples (200 µL) were drawn from the tail vein into heparinized microtubes after 12 h fasting period for different evaluations. At the end of 4 and 12 weeks the groups of animals were euthanized under deep anesthesia by single intraperitoneal overdose injection of sodium thiopental (150 mg/kg of body mass: Thiopentax^®^) followed by exsanguination by puncturing the abdominal aorta for plasma harvest and excision of footpads for the histopathological and mRNA expression analysis.

### Plasma CETP and lipoprotein level evaluation

The plasma CETP levels measured by ELISA (µg/ml ± SD) were 5.80 ± 2.07 and 0.02 ± 0.02 for CETP-Tg and WT mice, respectively. Transgenic animals have plasma CETP concentrations similar to humans (13). CETP activity, was measured using exogenous substrates as previously described by Cazita et al. (12). Cholesterol and triglyceride levels were evaluated by commercial enzymatic kits (RX series, Randox Laboratories).

### Parasites

*Leishmania (L.) amazonensis* (MHOM/BR/1973/M2269) promastigotes were grown at 26°C in M199 medium (Sigma) supplemented with heat-inactivated 10% Fetal Bovine Serum (FBS) (Gibco) until the stationary growth phase.

### Experimental design and lesion kinetics

8–12-weeks-old male mice from each experimental group were infected in the right hind footpads with 1 x 10^7^ stationary phase *Leishmania L. amazonensis* promastigotes. As a control, RPMI medium was injected in the left hind footpads. In the C57BL/6 mice the lesion size peaked at seven weeks post-infection (PI) and healed between 8–12 weeks (18).

The lesion progression was monitored over the course of a 12-week post infection by measuring the both footpad thickness with a digital caliper with an accuracy of 0.01 mm (Kingtools^®^). The size of the lesion was expressed by the difference between the infected and the non-infected footpads. Groups of animals were sacrificed at 4 and 12 weeks (7–8 animals/group) and, at the end of each experimental period, footpads were removed and processed as described below for histopathological and mRNA expression analysis.

### Histopathological analysis

The histopathological analysis of the infected and uninfected footpad from the animals of each group were processed and fixed in 10% phosphate-buffered formalin for subsequent embedment in paraffin. Sections (4 μm) were cut on a microtome (Carl Zeiss Hyrax M25) and stained with Hematoxylin-Eosin (HE). The nature of the inflammatory infiltrate and the presence of parasite forms were determined. Photomicrographs were taken on an image-capturing microscope (Leica DM5500B).

### Immunohistochemistry

Immunohistochemical reactions were performed with modifications (19, 20), using the antibodies listed in **Table 1**.

**Table 1:** Primary antibodies and their respective dilutions used in immunohistochemistry reactions.

Antibodies	Dilution	Description/Brand
**CD68** Mouse Monoclonal (Clone PG-M1)	1/100	Dako/M0876
**CD163** Rabbit Polyclonal	1/100	Spring/E18684
**CD206** Mouse Monoclonal	1/1000	BioRad/MCA55527
**iNOS** Rabbit Polyclonal	1/200	SC-651 – Santa Cruz Biotechnology
**ARG1** Rabbit Monoclonal	1/400	Arginase Sigma HP A003595 lote c60727
**CETP** Anti-Human Rabbit Polyclonal	1/200	CETP (H-300): sc-25833 Santa Cruz
**Anti-*Leishmania***	1/100	Serum from naturally infected dogs

The positive cells were quantified according to the antibody used. Ten fields were randomly chosen, and the image captured using a light microscope (Carl Zeiss), with 100X magnification, and AxioVision 4.8.2 software (Carl Zeiss). The positive cells were counted in each of the 10 fields with Image J software and data was presented as cell density (number of cells/mm^2^).

### RNA isolation and real-time PCR

Total RNA from 6–12 paraffin-embedded sections, 10 µm each, was extracted using both RNeasy FFPE kit (Qiagen) and Deparaffinization Solution (Qiagen), according to the manufacturer’s instructions. After elution, the RNA purity and concentration were measured in spectrophotometer Nanodrop ND-2000 (Thermo Fischer Scientific). cDNA was synthesized from 800 ng of total RNA with High- Capacity cDNA Reverse Transcription Kit (Applied Biosystems), according to the manufacturer’s instructions, in a final 40 µL reaction volume. The reactions were performed using: 1X HOT FIREPol EvaGreen qPCR Mix Plus (ROX) (Solis Biodyne), 250 nM of each of the primers (**Table 2**), 1 µL of cDNA (20 ng) and ultrapure water for a 20 µL final volume in triplicates in the thermocycler StepOne Real-time PCR System (Applied Biosystems). The amplification conditions were the same for all primers, as following: initial denaturation at 95°C for 15 min, amplification in 40 cycles of 95°C for 15 seconds, 59°C for 30 seconds and 72°C for 30 seconds. The relative expression of the genes were calculated using β- actin as a housekeeping gene according to the formula 2^−ΔΔCT^ target/2^−ΔΔCT^ β-actina (21).

**Table 2:** Sequences of primers used to evaluate gene expression in the lesion of mice infected with *L. (L.) amazonensis* by qPCR.

Target gene	*GenBank accession number*	Primer sequence
**Beta-actin**	NM_007393.5	F: GCCTTCCTTCTTGGGTATGGAATC R: ACGGATGTCAACGTCACACTTCAT
**CETP**	NM_000078.3	F:CAGATCAGCCACTTGTCCAT R:CAGCTGTGTGTTGATCTGGA
**SRA**	NM_031195.2	F: TACAGCAAAGCAACAGGAGGACA R: TGCGCTTGTTCTTCTTTCACAGAC
**LOX-1**	NM_138648.2	F: TCTTTGGGTGGCCAGTTACTACAA R: GCCCCTGGTCTTAAAGAATTGAAA
**ABCA1**	NM_013454.3	F: GAAGTTGGCAAGGTTGGTGAATG R: GGTTCATCCAGAAACACCACAGG
**LDLR**	NM_010700.3	F: AACCTGAAGAATGTGGTGGCTCTC R: CATCAGGGCGCTGTAGATCTTTTT
**CD36**	NM_001159558.1	F: GGCTAAATGAGACTGGGACCATTG R: AACATCACCACTCCAATCCCAAGT

### Lipoprotein profile

The plasma lipoprotein profile was determined by gel filtration chromatography (FPLC) in the Superose 6HR 10/30 column (GE Healthcare) coupled in the AKTA Purifier Liquid Chromatography System (GE Healthcare). A volume (100 µL) of a plasma pool obtained from the animals in each group was injected and elution occurred at a constant flow of 0.5 mL/min with Tris buffer (10 mM Tris, 150 mM NaCl, 1 mM EDTA and 0.03% NaN3, pH 7.0). After the collection of fractions, total cholesterol and triglycerides were determined by enzymatic-colorimetric method using Labtest kits (Labtest Diagnóstica). The identification of peaks corresponding to VLDL, LDL and HDL lipoproteins was determined by the absorbance of total cholesterol.

### Isolation of bone-marrow-derived macrophages

Bone marrow-derived macrophages (BMDM) were aseptically harvested from WT or CETP-Tg mice after euthanasia and were extracted from the femur and tibia bones. The cell suspension was centrifuged at 1200RPM for 10 min, the pellet obtained was resuspended in RPMI 1640 medium supplemented with 10% FBS, 30% LCCM (L-929 cell conditioned medium), 100 U/mL penicillin, 100 mg/mL streptomycin, 2 mM glutamine and distributed and maintained in polystyrene petri dishes (BD Biosciences) for 7 days at 37°C in a humid atmosphere with a 5% CO_2_. On the fourth day, an additional 10 mL RPMI 1640 medium supplemented with 10% FBS and 30% LCCM per plate were added.

### Infection of BMDM with *L. amazonensis*

BMDM (5x10^5^) were dispensed (100µl) onto round 13-mm glass cover slips, which were placed in 24-well plates (Corning Costar, USA) and incubated for 24h at 37°C in a humid atmosphere with 5% CO_2_ to allow adhesion. The wells were washed twice with culture medium to remove non-adherent cells. Then, the promastigote suspensions (five parasites per cell) were dispensed into the wells, and infection was allowed to occur for 4 hours at 32°C in a humid atmosphere with 5% CO_2_. After incubation, the non-internalized parasites were washed away. The culture was then maintained for 24, 48 and 72 hours at 37°C in a humid atmosphere with 5% CO_2_. The slides were stained with Panotico (Panoptica Rápido, Laborclin). The evaluation of parasitism under light microscope (Carl Zeiss, Gottingen, Germany) counting 300 cells per each treatment condition. The data were presented as the number of parasites per 100 cells from the formula [(number of parasites/number of infected cells) x (number of infected cells/total number of cells) x100] (22).

### NO production

Nitrite (NO_2_) accumulation in the cell culture supernatants was used as an indicator of NO production and was determined using the standard Griess reaction (23).

### Arginase activity

Cells and infected cells were removed from culture, lysed and used to determine arginase activity according to Corraliza et al. (24).

### Statistical analysis

All statistical analyses were performed using GraphPad Prism 9 (GraphPad Software, La Jolla, CA, USA). Data are expressed as mean ± standard deviation of the mean (SD). The Kruskal-Wallis test followed Dunn's post-test was applied for multiple comparisons. For comparisons between two unpaired groups, the Mann-Whitney test was used. p-values < 0.05 were considered statistically significant.

## Results

### CETP mice show improved resolution of *Leishmania (L.) amazonensis* infection

**Figure 1:**
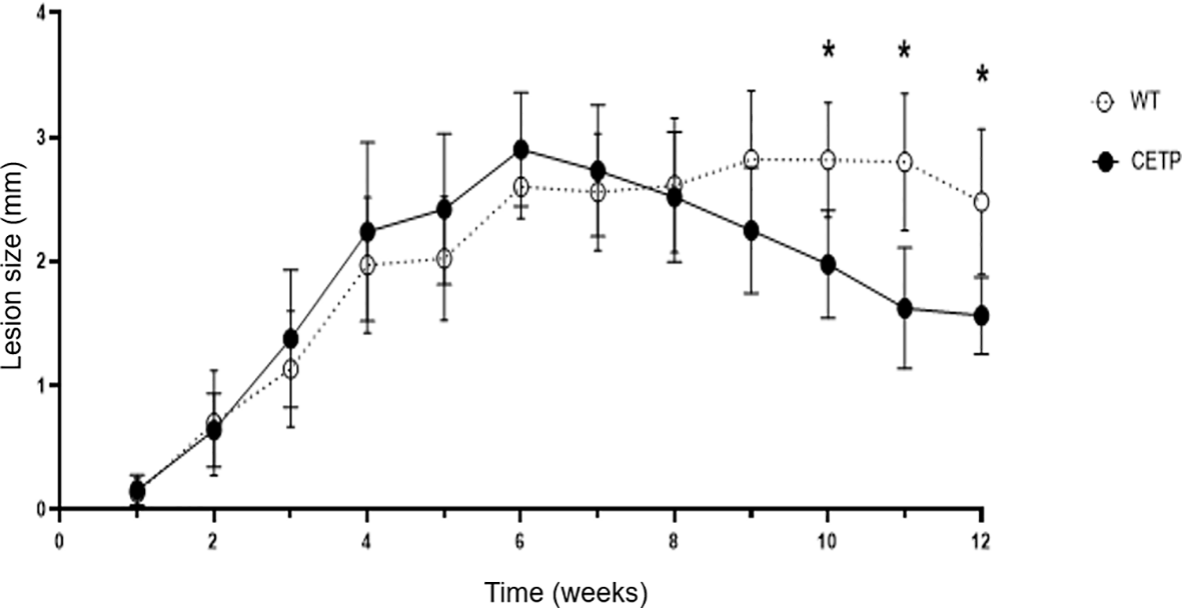
Lesion evolution in WT and CETP mice infected with *L. (L.) amazonensis* for 12 weeks post-infection (PI). Weekly assessment of the lesion size of the animals’ footpad considering the difference between the thickness of the infected and uninfected paw. Results expressed as mean ± SD of three independent experiments (n= 12), compared by Kruskal Wallis test, followed by Dunn’s test: WT *vs.* CETP 12 weeks PI; * *p*<0.01.

**Figure 2:**
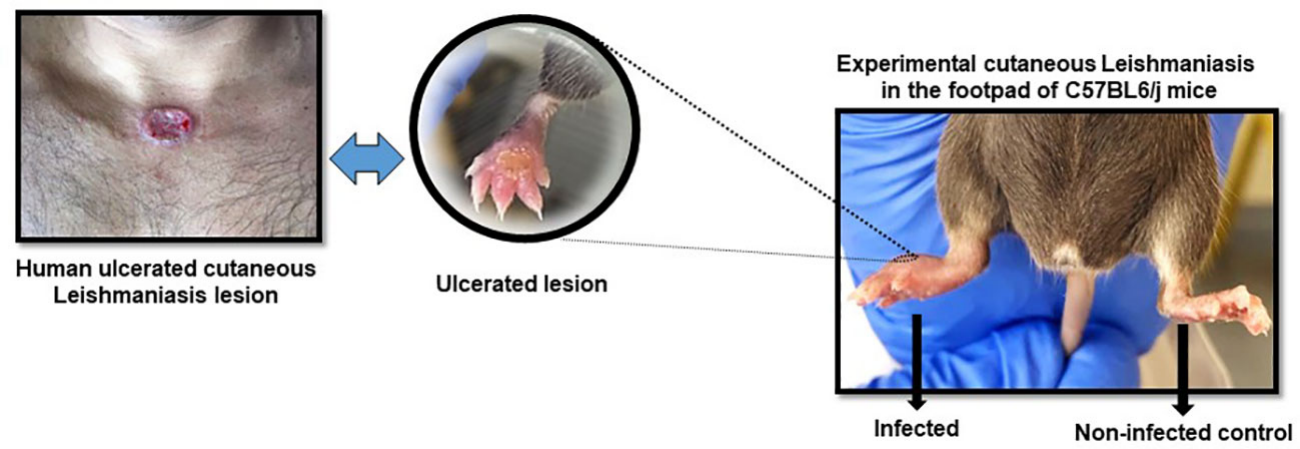
Representative figure of cutaneous leishmaniasis lesion in animals in the 6th week post-infection with *L. (L) amazonensis* promastigotes. Infected right footpad showing ulceration and contralateral footpad as control.

Progressive dermal lesion in response to *L. (L.) amazonensis* infection showed an increase from the 4th to the 12th week post-infection (PI) as compared with the 1st week, in both groups of WT and CETP animals (Kruskal-Wallis, p < 0.0001; Dunn, p ≤ 0.001) **Figure 1**. The lesion reached its peak development in the 6th week with a mean lesion thickness of 2.90 millimeters (mm) in the CETP compared with 2.61 mm in the WT group. Subsequently, approximately 90% of the animals developed ulceration in both groups (**Figures 1**, **2**). From the 9th week onwards, the lesion gradually diminished in the CETP animals, presenting smaller lesions from the 10th to the 12th week in relation to the WT group (p ≤ 0.01) (**Figure 1**). Upon completion of twelve weeks, in the CETP group, it was observed a diminished lesion with an average thickness of 1.56 mm of the infected paw lesion, similar to the thickness at 3rd week, while in the WT group it was observed ulceration and lesion with an average thickness of 2.48 mm (**Figure 1**).

### Histopathological analysis

**Figure 3:**
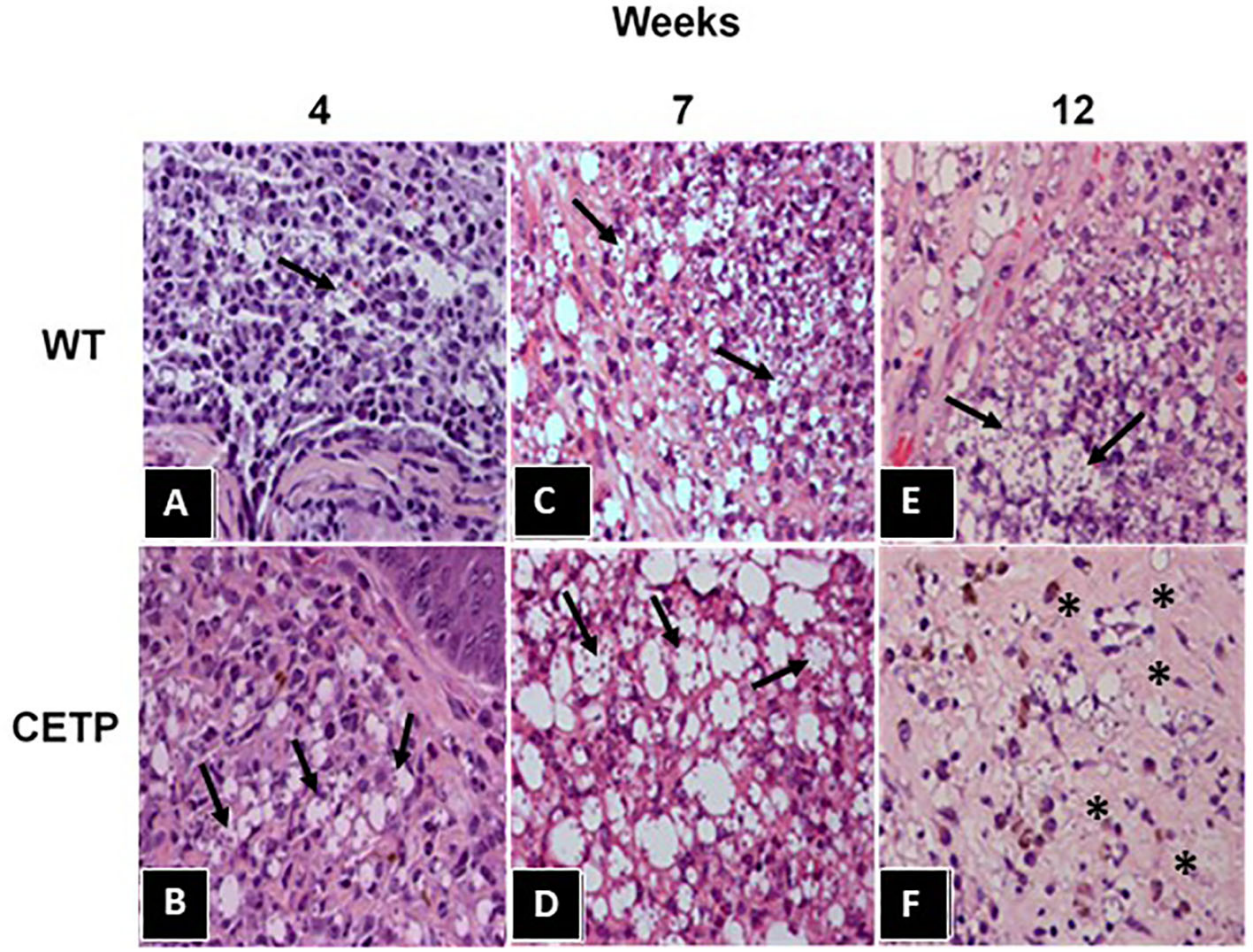
Histopathological analysis from footpad lesions of WT and CETP mice at 4, 7 and 12-weeks post-infection with *L. (L.) amazonensis.* Hematoxylin-Eosin (HE) staining. WT **(A, C, E)** and CETP **(B, D, F)**. Representative images from 3 independent experiments. Magnification 40x. Asterisk indicates presence of fibrosis and arrows indicate the presence of amastigotes within macrophages.

In histopathological analyses, with hematoxylin and eosin staining (HE), in the 4th week, we observed an area of extensive and diffuse lesion, with a predominance of vacuolated macrophages and intense parasitism, in both experimental groups (**Figure 3**), especially in CETP animals (**Figure 3B**) compared with WT (**Figure 3A**). A subgroup of animals (total 6 CETP and 6 WT animals, in two experiments) was euthanized in the 7th week, revealing a predominance of mononuclear cells and an increased number of macrophages in the lesions of both groups (**Figures 3C, D**). Notably, macrophages in CETP mice exhibit larger parasitophorous vacuoles (**Figure 3D**) compared with WT mice (**Figure 3C**). At 12 weeks, the CETP group showed decrease in inflammation and parasitism, the presence of fibrosis interstitial scar in both the superficial and deep dermis, accompanied by a mild mononuclear infiltrate, absence of giant cells and complete absence of parasitized macrophages (**Figure 3F**). In contrast, the WT group exhibited vacuolated macrophages and numerous forms of amastigotes (**Figure 3E**).

**Figure 4:**
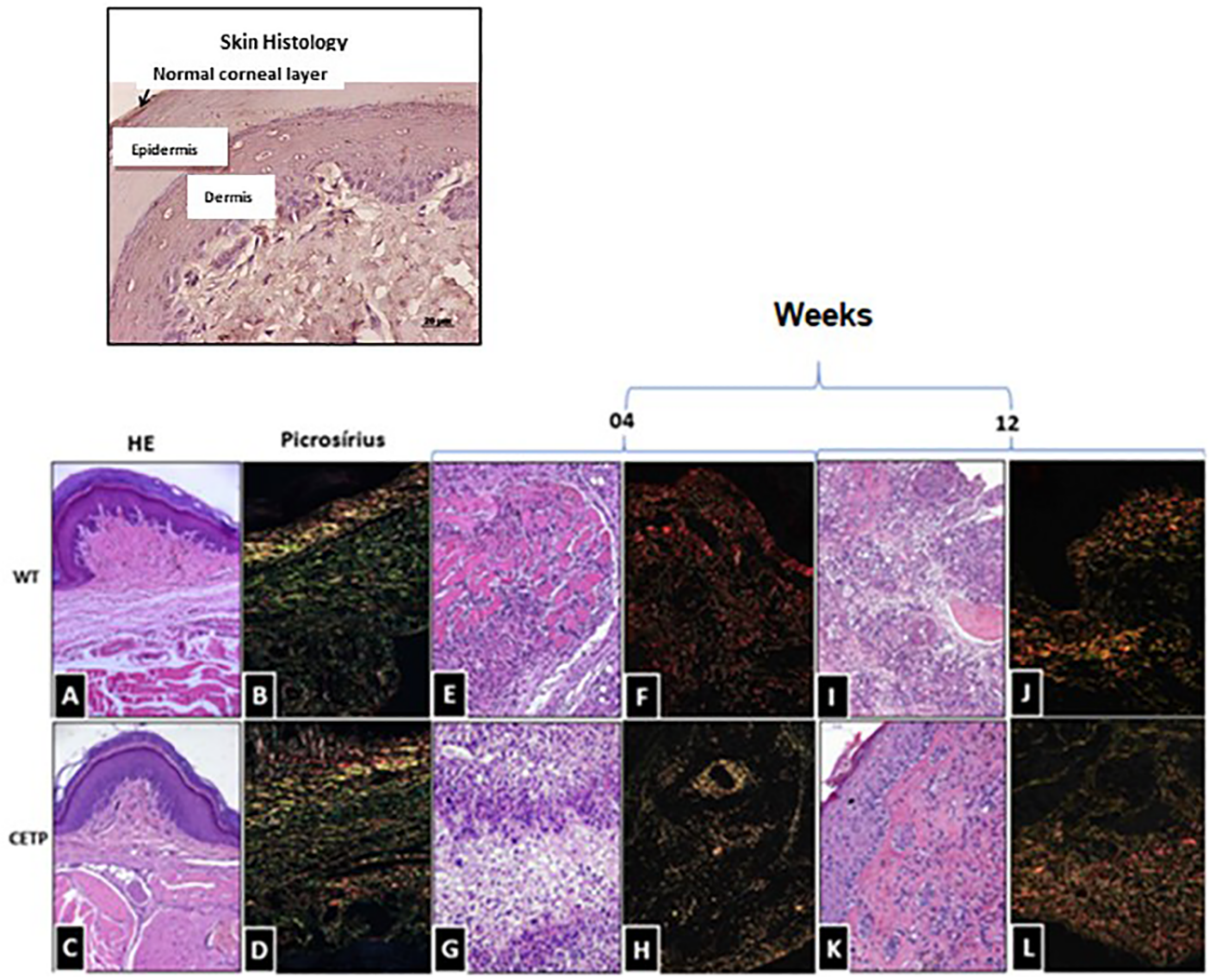
Histopathological analysis of collagen fibers in footpad lesions of WT and CETP mice at 4 and 12-weeks post-infection with *L. (L.) amazonensis*. HE staining in WT **(A, E, I)** and CETP **(C, G, K)**; Picrosirius staining in WT **(B, F, J)** and CETP **(D, H, L)** in the 4th and 12th week PI analyzed under an optical microscope (20x magnification). Representative images from three independent experiments.

Picrosirius staining of the mouse lesions confirmed the integrity of the tissue in the uninfected control footpad (**Figures 4A, C**). In infected animals, we observed a marked presence of type 1 collagen in both groups (**Figures 4B, D**). However, at week 4, both groups exhibited chronic inflammation and an area of necrosis (**Figures 4E, G**).

Notably, in the WT group, a dense layer was identified by Picrosirius (red), associated with collagen fragmentation (**Figure 4F**). At the end of the 12-week, CETP animals showed reduced parasitism, decreased inflammation and increased fibroblasts, indicative of collagen remodeling and progress in the healing process (**Figures 4H, K, L**). WT mice still showed parasitism, inflammatory infiltrate, tissue degeneration, necrosis, with the presence of macrophages displaying large vacuoles and collagen fragmentation (**Figures 4I, J**).

### CETP animals show reduced parasitism in the lesion and macrophage polarized towards M2-type

**Figure 5:**
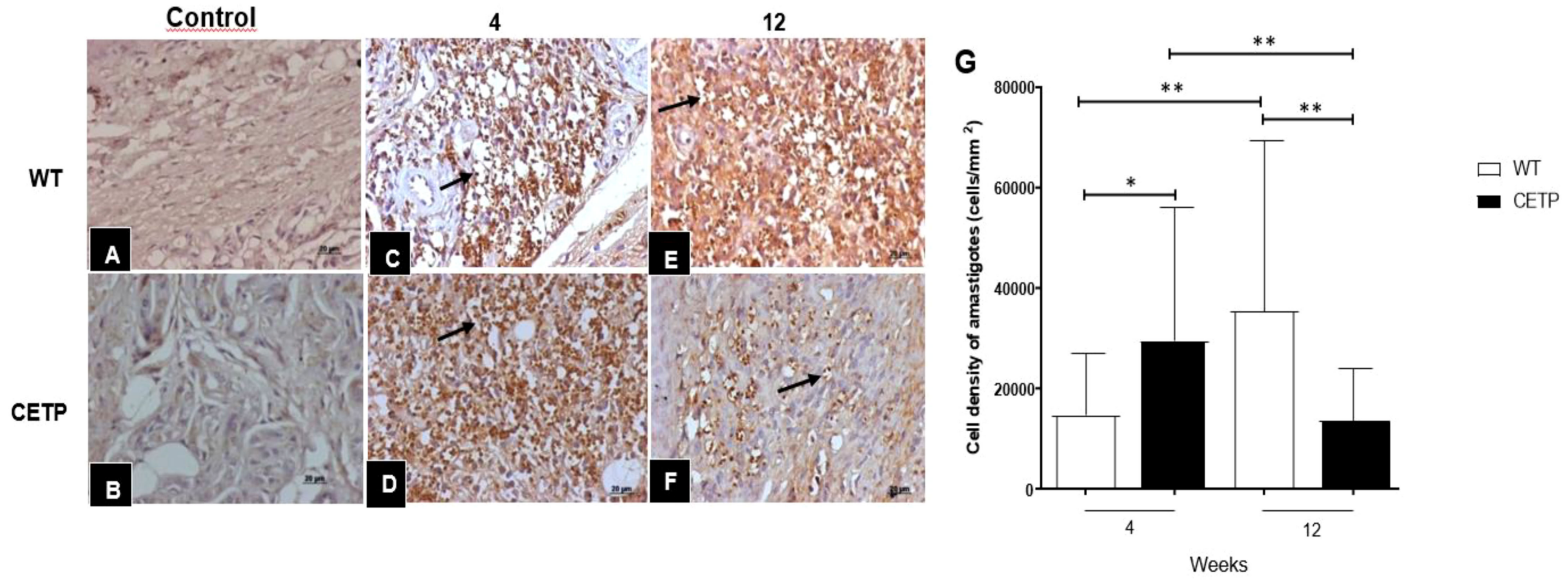
Histopathological analysis of parasitism in footpad lesions of WT and CETP mice at 4 and 12-weeks post-infection with *L. (L.) amazonensis*. Immunostaining with anti-*Leishmania* polyclonal antibody. Control, uninfected footpad: WT **(A)** and CETP **(B)**. Infected: WT **(C, E)** and CETP **(D, F)** in the 4*th* and 12*th* week PI analyzed under an optical microscope (100x magnification). Representative images from three independent experiments. Arrows indicate the presence of amastigotes within the parasitophorous vacuoles of macrophages. **(G)** Representative graph of amastigote cell density (cells/mm2). Results expressed as mean ± SD of two independent experiments, n= 4–6 per group, compared by Mann-Whitney test: WT *vs.* CETP 4th and 12th weeks PI; * *p*<0.05 and ** *p*<0.005.

At 4 weeks PI, the CETP group showed an increase in parasitism represented by the cell density of amastigotes (**Figures 5D, G**) compared with the WT group (**Figures 5C, G**). However, the progression of the lesion over 12-weeks post- infection, showed an inversion in the response, where the CETP animals presented a lower density of amastigotes in the lesions (**Figures 5F, G**) compared with the WT group (**Figures 5E, G**) and the 4-week period (**Figure 5D**). The WT animals showed an increase in parasitism at 12 weeks (**Figures 5E, G**). Uninfected control paws from WT and CETP animals are represented in **Figures 5A, B**, respectively.

**Figure 6:**
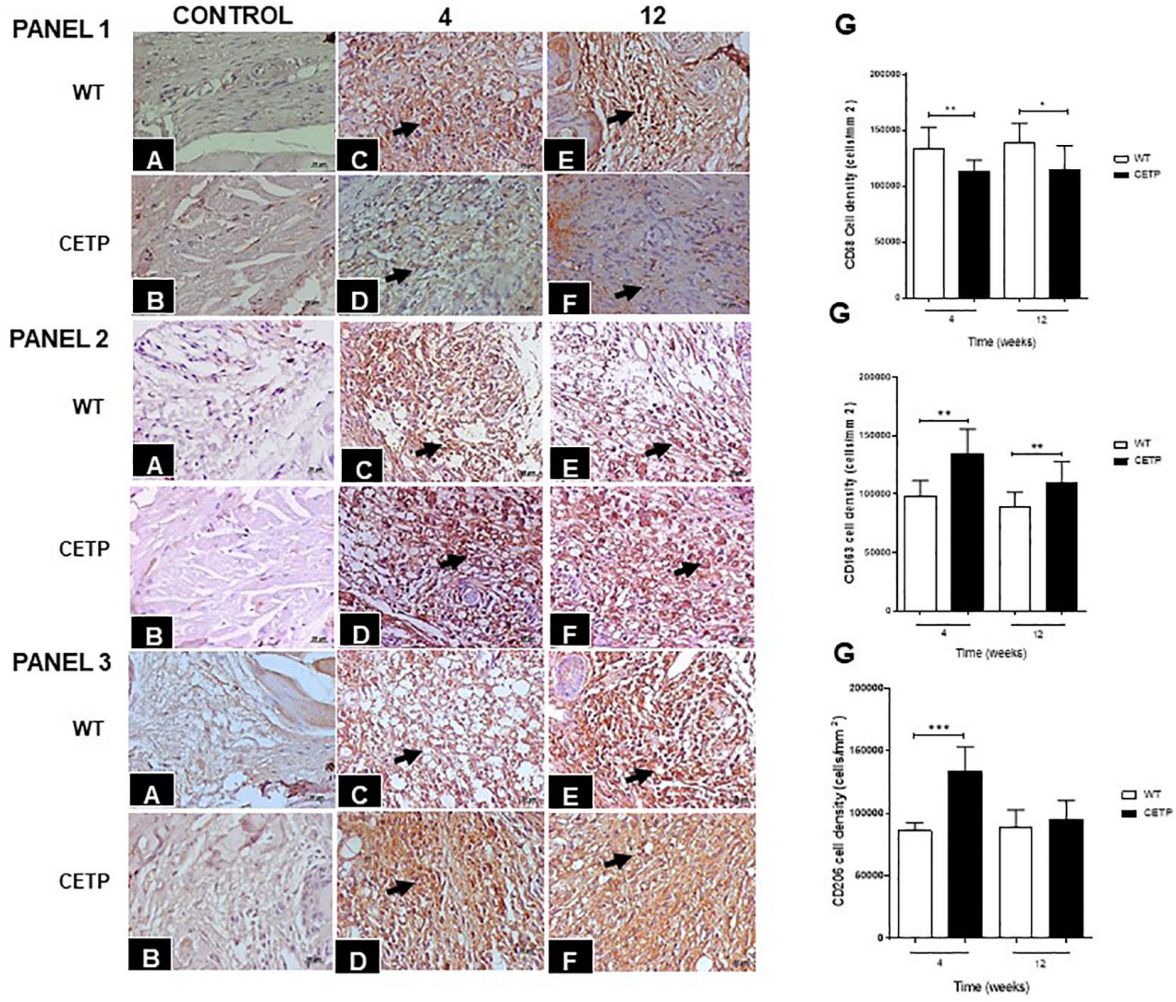
Histopathological analyses of CD68+ (PANEL 1), CD163+ (PANEL 2), and CD206+ (PANEL 3) cells in footpad lesions of WT and CETP mice at 4 and 12-weeks post-infection with *L. (L.) amazonensis*. Control, uninfected footpad: WT **(A)** and CETP **(B)**. Infected: WT **(C, E)** and CETP **(D, F)** in the 4th and 12th week PI analyzed under an optical microscope (100x magnification). Representative images from three independent experiments. Arrows indicate the presence of immunostaining in macrophages. **(G)** Representative graph of cell density (cells/mm2) with the respective macrophage markers. Results expressed as mean ± SD, n= 4–6 per group, compared by Mann-Whitney test: WT *vs.* CETP 4th and 12th weeks PI; * *p*<0.05 and ** *p*<0.005, ***p<0.001.

Infection with *L. (L.) amazonensis* resulted in an increase in CD68+ cells in the dermal inflammatory infiltrate (**Figure 6, panel 1**). The density of CD68+ cells was lower in CETP mice (**Figure 6, panel 1D**) compared with the WT group at 4 and 12 weeks (**Figure 6, panels 1C, E–G**). The number of CD163+ cells, visualized in the inflammatory infiltrate of the lesions, was higher in the CETP group compared with the WT, at week 4 and 12 (**Figure 6, panels 2C–G**). There was an increase in the density of CD206+ cells only at week 4 in the CETP group, with no differences between the groups at 12 weeks (**Figure 6, panels 3C–G**). Uninfected control paws from WT and CETP animals are represented in **Figures 6A, B**, respectively in all panels (1, 2 and 3).

**Figure 7:**
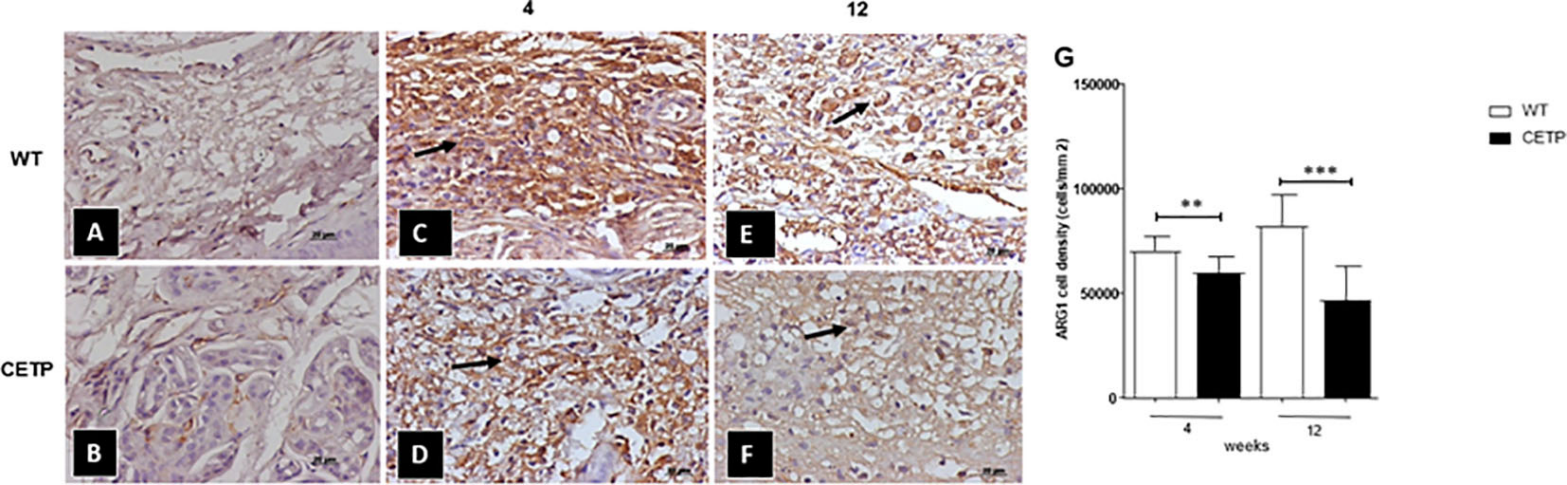
Histopathological analysis of arginase cells (ARG 1+) in footpad lesions of WT and CETP mice at 4 and 12-weeks post-infection with *L. (L.) amazonensis*. Control, uninfected footpad: WT **(A)** and CETP **(B)**. Infected: WT **(C, E)** and CETP **(D, F)** in the 4th and 12th week PI analyzed under an optical microscope (100x magnification). Representative images from three independent experiments. Arrows indicate ARG1+ cells. **(G)** representative graph of ARG 1+ cell density (cells/mm2). Results expressed as mean ± SD, n= three per group, compared by Mann-Whitney test: WT *vs.* CETP 4th and 12th weeks PI; ** *p*<0.006 and ***p<0.0001.

**Figure 8:**
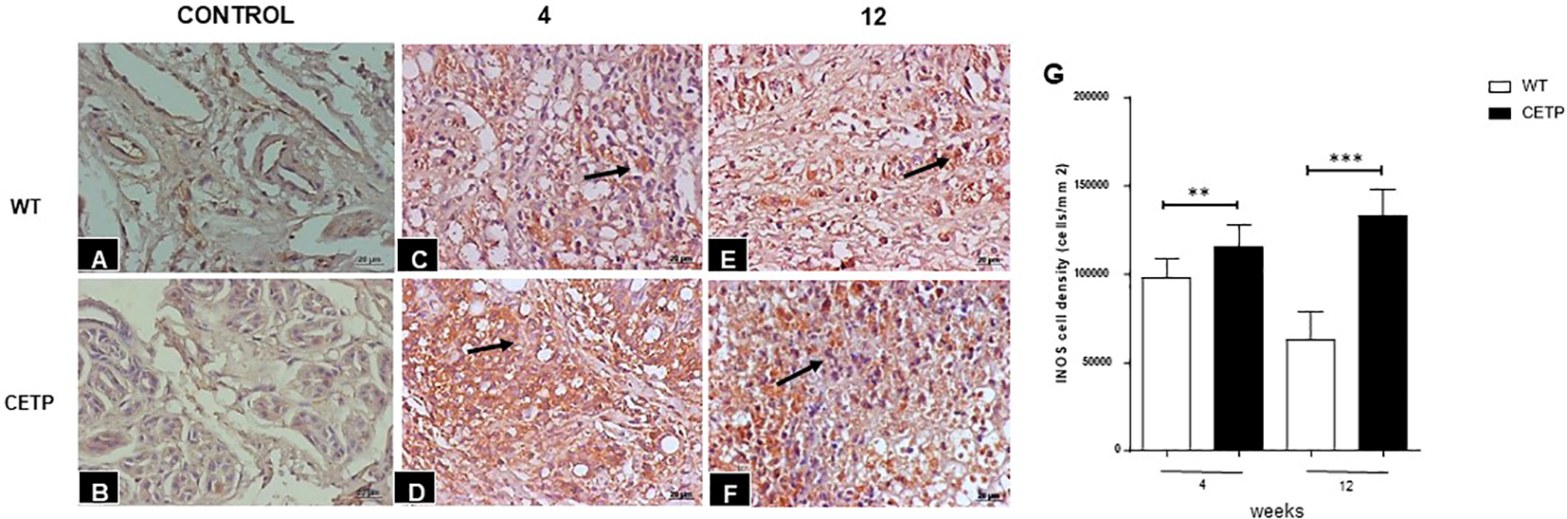
Histopathological analysis of cells iNOS+ in footpad lesions of WT and CETP mice at 4 and 12-weeks post-infection with *L. (L.) amazonensis*. Control footpad without infection: WT **(A)** and CETP **(B)**. Infected: WT **(C, E)** and CETP **(D, F)** in the 4th and 12th week PI analyzed under an optical microscope (100x magnification). Representative images from three independent experiments. Arrows indicate iNOS+ cells. **(G)** iNOS cell density (cells/mm2). Results expressed as mean ± SD, n= three per group, compared by Mann- Whitney test: WT *vs.* CETP 4th and 12th weeks PI; **p<0.004 and ***p<0.0001.

We observed a reduction of ARG1+ cells in the CETP compared with the WT group at 4 and 12 weeks (**Figure 7**). However, the density of iNOS+ cells showed an increase in the CETP lesion (**Figures 8D, F, G**) compared with the WT at 4th and 12th weeks PI (**Figures 8C, E, G**). Uninfected control paws from WT in **Figure 8A** and CETP **Figure 8B**.

**Figure 9:**
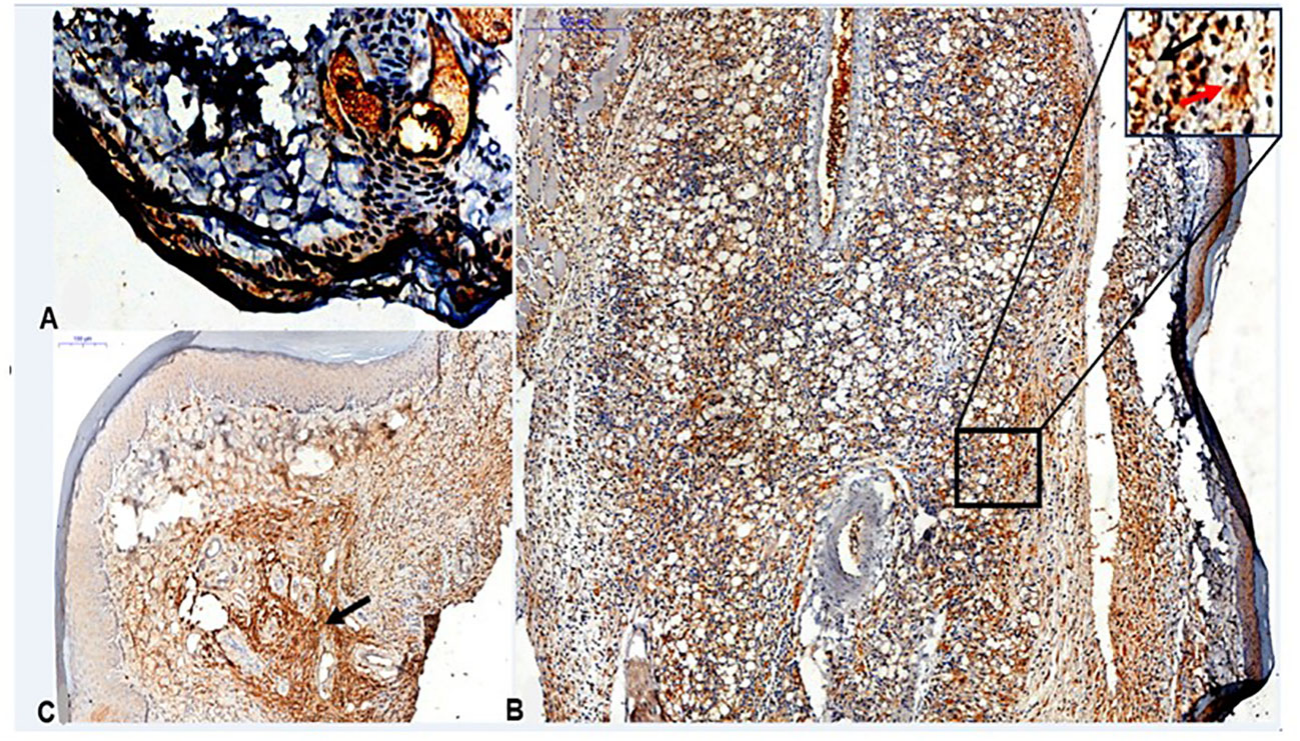
Histopathological analyses of CETP+ cells in footpad lesions of CETP mice at 4 and 12-weeks post-infection with *L. (L.) amazonensis*. **(A)** Control, uninfected footpad (magnification 200x). **(B)** Infected in the 4th week PI and **(C)** infected in the 12th week PI analyzed under an optical microscope (50x magnification). Representative images from three independent experiments. CETP labeling in interstitial macrophages (in brown, by peroxidase). In detail, at the top right, it marks of uninfected macrophages, present in the interstitial inflammatory infiltrate (black arrow) and the presence of amastigotes within the parasitophorous vacuoles of macrophages (red arrow).

We identified the presence of CETP in the lesions of footpad of the infected transgenic animals in interstitial macrophages at 4th and 12th weeks PI (**Figure 9**).

**Figure 10:**
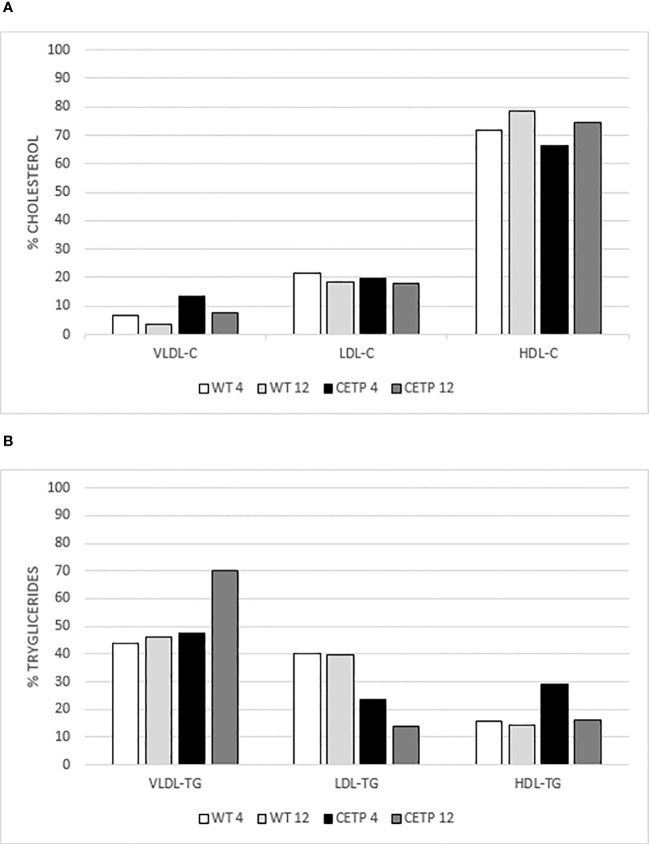
Analysis of the lipoprotein profile. Percentage of cholesterol (C) in **(A)** and triglycerides (TG) in **(B)** by gel filtration chromatography (FPLC). Plasma pool of WT or CETP mice at 4 and 12-weeks post-infection with *L. (L.) amazonensis.* VLDL: very low-density lipoprotein; LDL: low-density lipoprotein; HDL: high-density lipoprotein.

After 4 weeks, qualitative analysis of the lipoprotein (LP) profile (**Table 3**, **Figure 10**) revealed that the CETP mice exhibited HDL particles with reduced cholesterol, increased triglycerides (TG) and a higher concentration of VLDL-C compared with the WT group (**Figures 10A, B**). After 12 weeks of infection, the CETP mice showed an increase in the concentration of TG in VLDL and a reduction in LDL and HDL (**Figure 10B**), compared with the analysis carried out at week 4 post-infection. In contrast, the WT group exhibited few changes in plasma lipoprotein composition over the course of the infection, showing only a slight increase in HDL-C and a reduction in VLDL-C at 12 weeks (**Figure 10A**). There was no difference in CETP activity in the evaluated periods (**Table 3**).

**Table 3:** Cholesterol, triglycerides and CETP activity in plasma of WT and CETP mice 4 and 12 weeks PI with *L. (L.) amazonensis.*

weeks	n	WT	4	CETP	WT	12	CETP
Cholesterol (mg/dL)	*pool*	65		70	79		60
Triglycerides (mg/dL)	*pool*	50		61	50		84
CETP activity (%)	4			23,7±2,9			21,5±3,8

### Expression of genes involved in lipid metabolism

**Figure 11:**
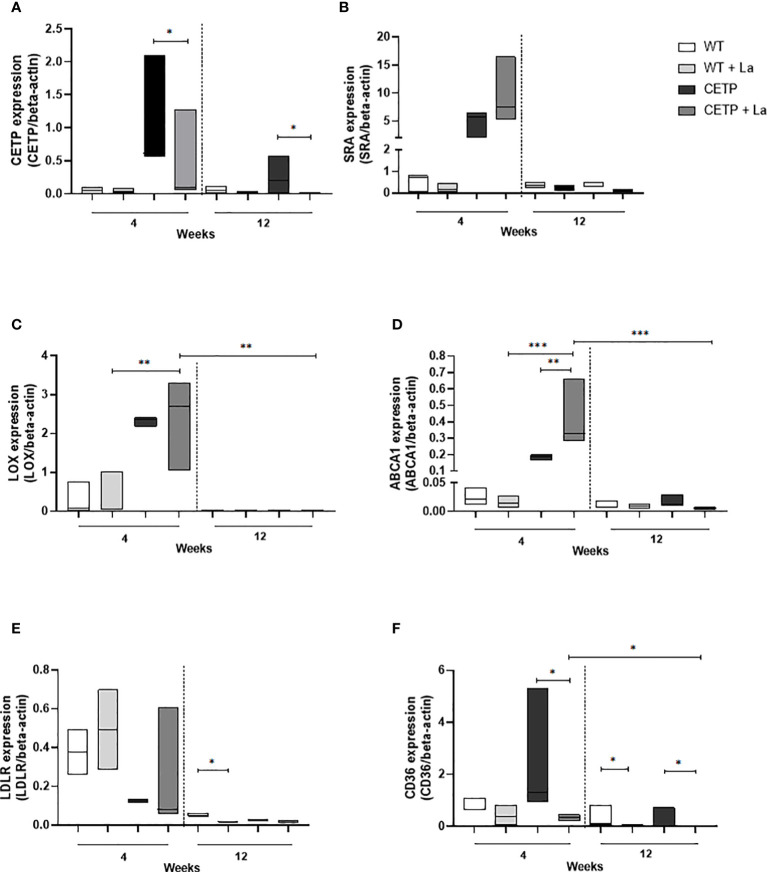
Gene expression in lesions of CETP transgenic mice and WT controls at 4 and 12-week post-infection with *L. (L.) amazonensis*. **(A)** CETP, **(B)** SRA, **(C)** LOX, **(D)** ABCA1, **(E)** LDLR, **(F)** CD36 mRNA. Data presented are expression levels of each target relative to beta-actin expression mRNA level. A representative experiment of two independent assays in triplicate. Results expressed as median ± SD, compared to the Kruskal-Wallis test: WT vs. CETP * p<0.05, **p<0.009 e ***p<0.0001.

We searched some genes related to lipid transport, some scavenger receptors shown in *Leishmania* infection models (25, 26). Lower expression of CETP mRNA was observed in the *Leishmania* infection in the 4th and 12th weeks compared with the control without infection (**Figure 11A**). No differences were identified in the expression of SRA receptor mRNA in either groups (**Figure 11B**). CETP animals showed higher expression of the gene encoding LOX in the presence of *Leishmania* at week 4 compared with infected WT. At 12 weeks, LOX mRNA expression was lower in the infected CETP compared to the 4th week (**Figure 11C**). There was an increase in ABCA1 mRNA expression in infected CETP animals compared with infected WT animals at week 4. This expression was higher than in the uninfected CETP group. At 12 weeks, ABCA1 mRNA expression was lower in the CETP group (**Figure 11D**). LDLR gene expression was lower in infected WT animals at week 12 compared to their controls (**Figure 11E**).

There was a reduction in the expression of the gene that codes for the CD36 receptor in the infected CETP animals compared with the uninfected, at week 4 and 12 (**Figure 11F**). Among the infected CETP group, there was a reduction at week 12 compared with week 4. The WT group showed a reduction in infected animals compared with non-infected animals at week 12 (**Figure 11F**).

### Parasitism, arginase activity and NO level in *L. (L.) amazonensis*-infected bone marrow cells *in vitro*

**Figure 12:**
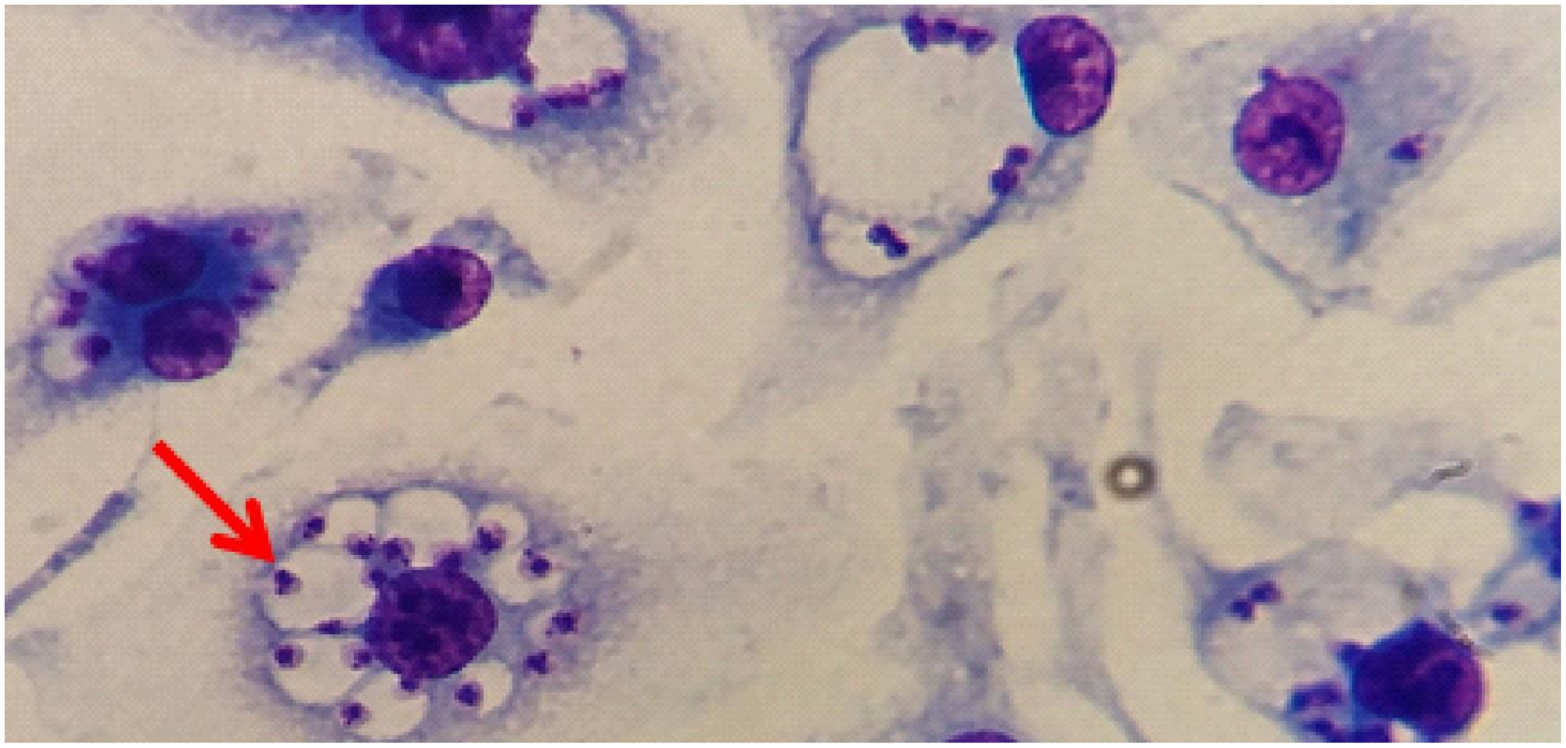
The presence of *L. (L.) amazonensis* in parasitophorous vacuoles of macrophages. Representative image of macrophages derived from bone marrow cells from C57BL6/J mice infected with *L. (L.) amazonensis* (red arrow) stained with Rapid Panoptic (Laborclin).

**Figure 13:**
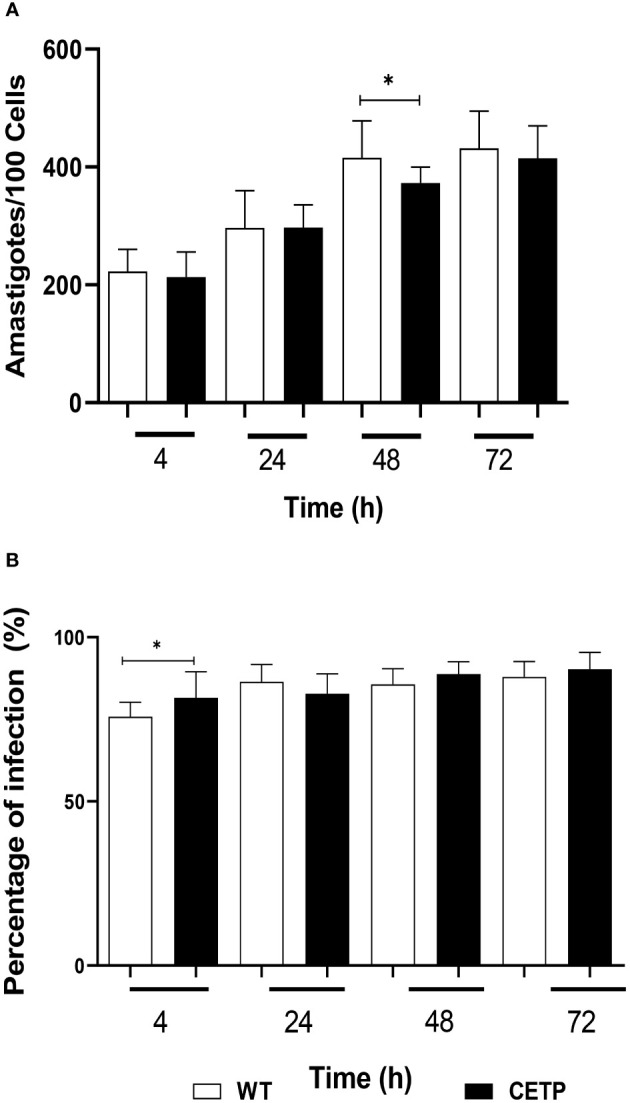
Parasitism of bone marrow-derived macrophages from WT and CETP mice infected with *L. (L.) amazonensis* promastigotes. Macrophages (5 x10^5^) were infected in a ratio of 5:1 (parasite: cell) for 4h of infection, followed by 24, 48 and 72h of incubation. **(A)** Parasitism is represented by the number of amastigotes/100 cells. **(B)** Percentage of infection. Results expressed as mean ± SD of three independent experiments in triplicate, compared by Mann-Whitney test: WT *vs.* CETP; * *p*<0.05.

**Figure 12** shows the effective infection with the presence of parasites in parasitophorous vacuoles of macrophages derived from bone marrow cells of mice infected with promastigote forms of *L. (L.) amazonensis*. A comparison of the parasitism of infected macrophages showed a linear increase over time. At 48h CETP cells showed a decrease in the number of parasites (373 parasites per 100 cells) when compared with 416 parasites in the WT cells (p=0.040) (**Figure 13A**). Analyzing the percentage of infection, CETP cells showed 84% at 4 h and WT cells, 76% (**Figure 13B**).

**Figure 14:**
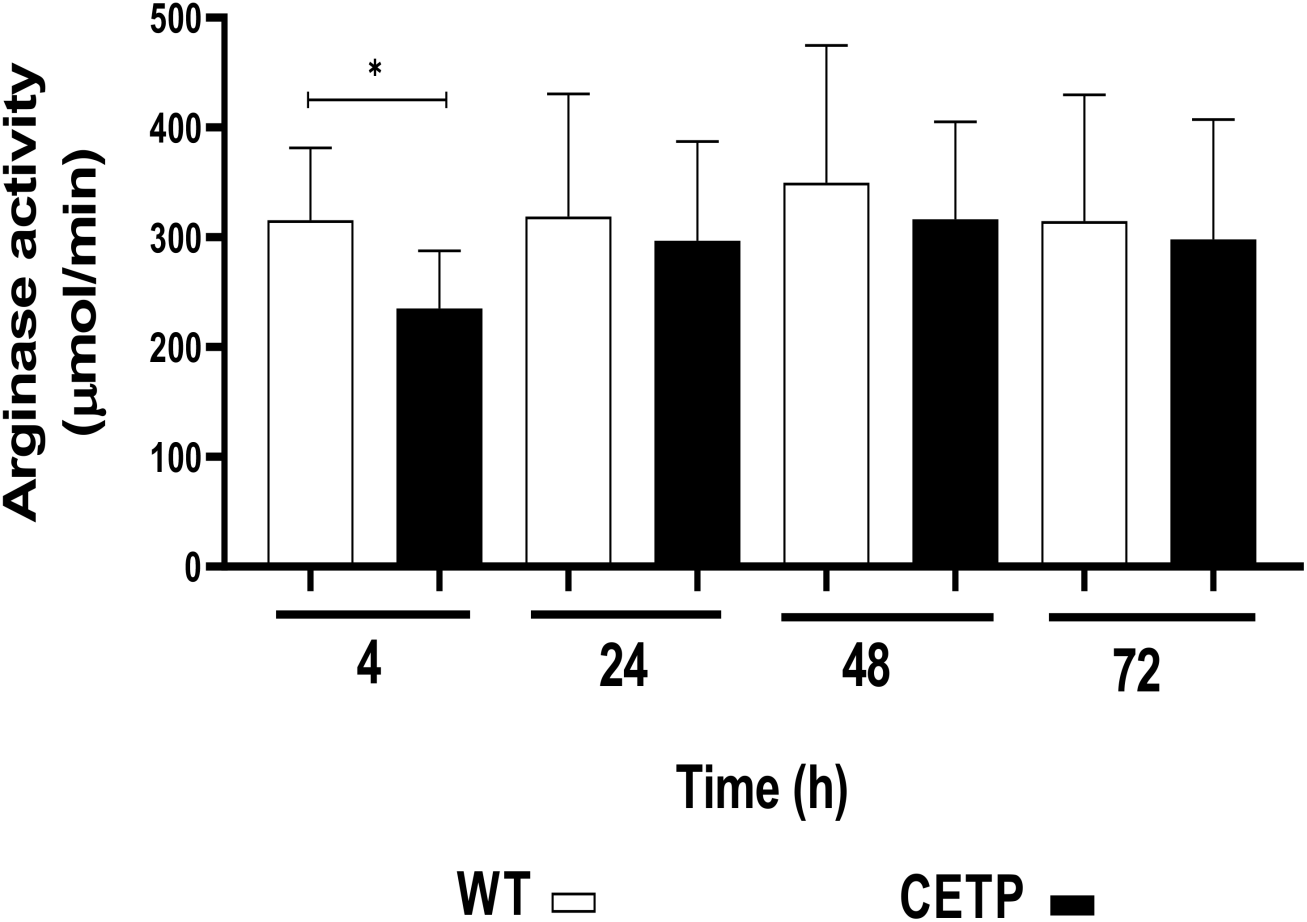
Arginase activity determined in the cell lysate of WT and CETP macrophages infected with *L. (L.) amazonensis.* Macrophages (5 x10^5^) were infected in a ratio of 5:1 (parasite:cell) for 4h of infection, followed by 24, 48 and 72h of incubation. Results expressed as mean ± SD of three independent experiments in triplicate, compared by Mann-Whitney test: WT *vs.* CETP. * *p*<0.05.

**Figure 15:**
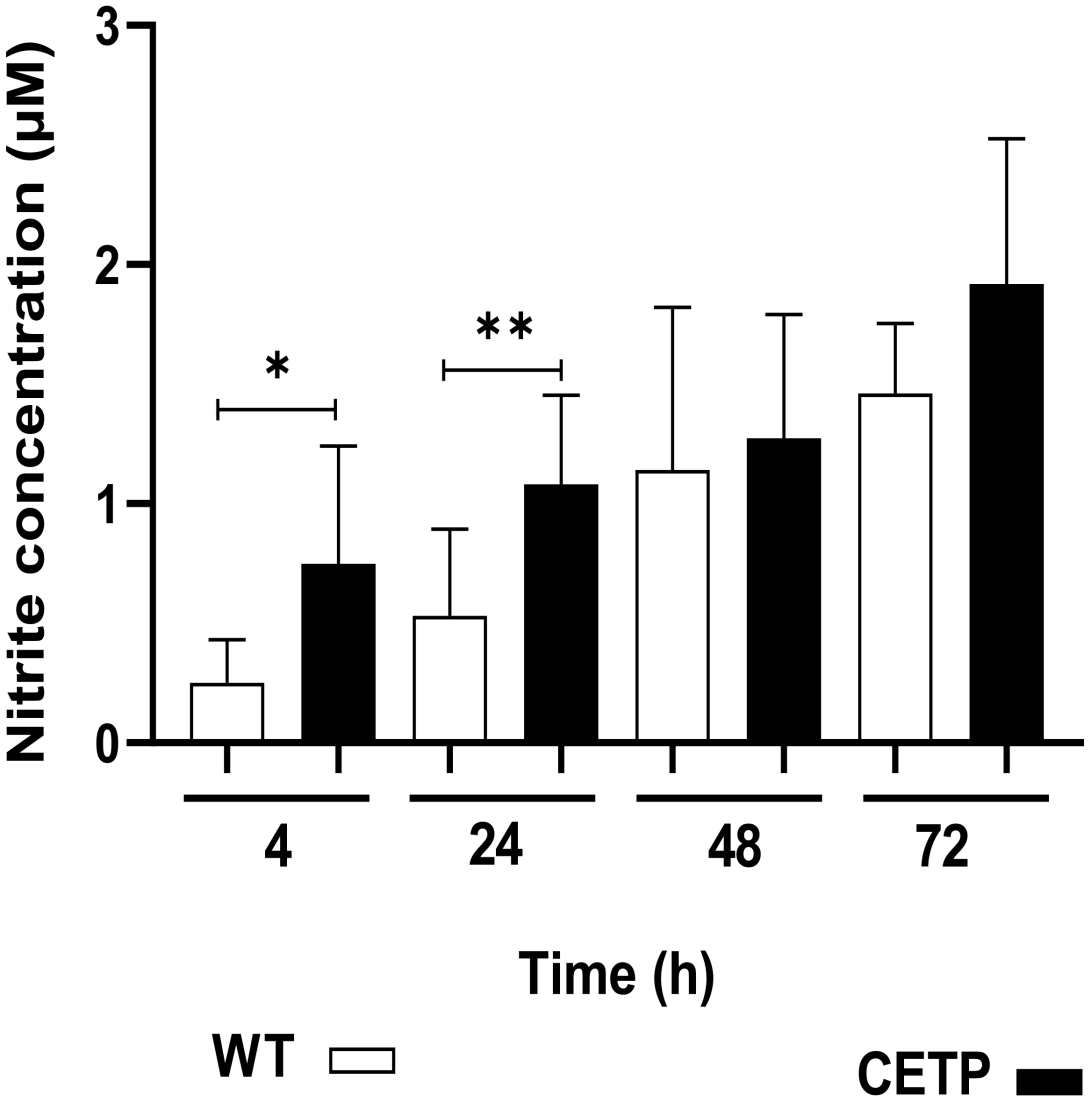
Nitric oxide production in the culture supernatant of bone marrow-derived macrophages from WT and CETP mice infected with *L. (L.) amazonensis.* Macrophages (5 x10^5^) were infected in a ratio of 5:1 (parasite:cell) for 4h of infection, followed by 24, 48 and 72h of incubation. Results expressed as nitrite concentration as the mean ± SD of three independent experiments in triplicate, compared by Mann-Whitney test: WT vs. CETP * p<0.05; ** p<0.01.

The CETP group, after 4 hours of culture compared with the WT group, presented lower arginase activity in 23% (p < 0.05) **Figure 14**. However, there was an increase in NO, determined by the concentration of nitrite, in the CETP group compared with the WT group at 4 and 24 hours, 166% and 100%, respectively (**Figure 15**).

## Discussion

In this present study, we investigated the impact of the expression of CETP, a protein that facilitates the transfer of lipids between LP, on the development of experimental CL in CETP-transgenic mice that express physiological plasma levels (similar to humans) of CETP and a slight reduction in HDL-C compared with littermates that do not express the protein, and also in cells derived from the bone marrow of these animals, compared with the control group.

In the first four to six weeks after the appearance of the skin lesion, the infected CETP animals showed an exacerbated response with a greater diameter of the lesion with raised edges, ulceration, and a necrotic background.

Subsequently, the dermal lesion regressed, and it was almost absent between the 10th and 12th week PI. In the WT group, dermal lesion formation was slower. The largest lesion size was reached at the 9th week. At the end of the analysis period, 12 weeks, parasitism and a moderate inflammatory process were observed, which may be related to the distinct immune response between the groups.

Considering the dynamics of the lesion and healing, the CETP group showed faster resolution compared with the WT group after the 10th week of infection. Scarring interstitial fibrosis and the absence of amastigotes in the CETP group after 12 weeks suggest better control of the infection upon analysis in HE and picrosirius-stained tissues.

Parasitism decreased significantly in the CETP group at week 12, not only compared with week 4 but also with WT animals, in which an increase in amastigotes was observed over the weeks.

It has been shown that tissue restoration is observed in the CL lesion by controlling the inflammatory process and accumulating collagen in the region of parasite elimination (18, 27, 28). In early remodeling and healing, type III collagen is first observed, which is later replaced by type I as the process consolidates (28, 29).

Our results are in line with these data and show that both types of collagen are present in the cushion lesions of both CETP and WT animals. However, the CETP group, after 12 weeks, showed remodeling and healing observed in the deeper layer, while the WT group, still in the inflammatory process, showed delayed infection development.

Other studies have shown that CETP-expressing mice, compared with control mice, notably have atherosclerotic lesions enriched in collagen, suggesting a role of CETP or diet in modifying the collagen content of the lesion (30). Further, the inhibition of CETP improved the recruitment of monocytes and the expression of MCP-1, producing lesions with marked inflammatory properties, as evidenced by the increase in macrophages and reduction in collagen content, reinforcing the anti-inflammatory role of CETP (31).

We recently showed that bone marrow macrophages from CETP mice polarize prone to an M2 profile after inflammatory stimulation with LPS (16). In the present study, specific markers for macrophage populations showed a diverse inflammatory response between the groups, with a reduction in CD68+ cells and an increase in CD163+ and CD206+ cells in the presence of CETP, showing polarization towards the M2 profile that is considered a pro-resolution response associated with subsequent stages of infection and inflammation control (32). Tissue recovery induces the migration of M2-type macrophage populations, which favors the migration and proliferation of keratinocytes, fibroblasts and other cell types that are important for restoring the dermis, epidermis and vasculature, in order to remodel the injured tissue (33).

Many genes, including the mannose receptor (CD206), arginase 1 (ARG1), heme receptor (CD163), and inducible nitric oxide synthase (iNOS), are involved in regulating the polarization of M1 and M2 macrophages (32). Macrophages resident in the liver (Kupffer cells) are reservoirs of the intracellular amastigote form in visceral leishmaniasis, characterized by the expression of characteristic macrophage markers (F4/80, CD14, CD68, CD11b). Kupffer cells maintain a distinctly anti-inflammatory environment, limiting antigen presentation capacity and exhibiting M2 profile characteristics (32). In addition, these cells are the main producers of CETP secreted into the circulation (34).

Considering that greater arginase activity promotes parasite survival, while nitric oxide (NO) production exhibits leishmanicidal action (35), the results of the present study, both *in vivo* and *in vitro*, corroborate these findings. There was a lower density of Arginase 1 positive cells in the CETP group compared with the WT animals, and an increase in the enzyme nitric oxide synthase (NOS) in its inducible isoform (iNOS) in the lesions, as evidenced by immunohistochemistry.

Under *in vitro* conditions, although the approach is restricted to an isolated cellular system disregarding the organism’s response, a reduction in arginase activity was observed in the CETP group compared with the WT group at 4 hours after infection. In addition, higher concentrations of nitrite (NO) were recorded in the cell cultures at the 4-hour and 24-hour intervals.

Anti-*Leishmania* activity is demonstrated for iNOS in skin biopsies collected from patients with CL, where the frequency of iNOS-positive cells had an inverse correlation with parasite load in CL lesions due to *L. Mexicana* (36, 37). In the present study, we observed an inverse correlation between the degree of parasitism and iNOS production (p=0.0368 r= - 0.4732). A positive correlation was also found between the degree of parasitism and arginase (p=0.0281 r=0.4904).

Changes in the expression of the LOX, ABCA1 and CD36 genes in the CETP mice indicate a possible association between lipid metabolism and the response to infection. An increase in the receptor for oxidized LDL (LOX) was observed in the CETP group infected in the 4th week compared with the WT group, correlating with parasitism during this period. Macrophages have LDL receptors that incorporate these particles under normal conditions. In pathological conditions, such as hyperlipidemia, genetic diseases and oxidative stress, specific components of LDL are oxidized, increasing their absorption. LOX expression was reduced at week 12 in the CETP group. The expression of the ABCA1 gene was positively regulated in the infected CETP animals, both compared with the uninfected CETP group at week 4 and the CETP group at 12 weeks, as well as showing an increase compared with the infected WT animals. These data suggest modulation of cholesterol traffic during *L. amazonensis* infection.

The cholesterol efflux activities mediated by ABCA1 and ABCG1 modulate the expression of inflammatory cytokines and chemokines by macrophages, as well as lymphocyte proliferative responses. In macrophages, the absence of the transporter results in increased signaling through various receptors, including TLR4, evidencing a connection between the traditional functions of HDL and ABCA1 transporters in the efflux and reverse transport of cholesterol with the anti-inflammatory and immunosuppressive functions of HDL. The underlying mechanisms may involve the modulation of sterol levels and lipid organization in cell membranes (38). Despite being secreted, it is possible that CETP also performs a local intracellular function, as suggested by cell culture studies manipulating CETP expression and the altered cholesterol traffic between membranes and accumulation of ester cholesterol and triglycerides (39, 40).

Despite the low expression of the CETP gene in the infected group when analyzed by qPCR, the immunohistochemistry shows the presence of the protein in the lesion at 4 and 12 weeks. Studies have revealed a temporary decrease in CETP gene expression in response to inflammatory stimuli, as evidenced by the response to LPS (41).

The CD36 receptor was negatively regulated in the presence of infection in the CETP group, correlating with healing and a decrease in parasite load. CD36 is associated with both the multiplication of amastigotes within the macrophage and the retention of cholesterol, as reported (25).

A study that analyzed the role of CD36 in mammalian models of *L. amazonensis* infection found that CD36 is concentrated in the PV membrane juxtaposed to the posterior end of amastigotes. Furthermore, the PV in macrophages from CD36−/− mice was reduced in size, as a consequence of reduced fusion of the late endosome and/or lysosomes with the PV, and did not support amastigote replication. Collectively, these studies identify an essential role for CD36 in PV maturation and Leishmania survival (42).

CETP is known to affect the composition of plasma LP and may influence the immune response. A study of patients with VL showed high TG concentrations and lower cholesterol at the time of diagnosis. HDL-C, apoA-I, and associated enzymes remain low four months after the resolution of the disease compared to controls (43). A study of hamsters with VL infected with *L. (L.) infantum* showed alterations in lipid metabolism with an increase in TG and a reduction in mRNA expression for CETP and lipoprotein lipase (44). The CETP mice exhibited HDL particles with reduced cholesterol, increased triglycerides (TG) and higher VLDL- TG concentration compared to the WT group in the presence of infection, although with no difference in CETP activity. The WT group exhibited few changes in the composition of plasma lipoproteins throughout the course of the infection, showing only a slight increase in HDL-C and a reduction in VLDL-C at 12 weeks. CETP has a clearly defined function in regulating cholesterol flow between plasma lipoproteins, leading to a decrease in HDL cholesterol, often associated (or not) with an increase in non-HDL cholesterol. Because of this effect, CETP inhibition has been considered a therapeutic target with anti- atherogenic potential. In addition, experimental and human studies have implicated CETP in protection against acute inflammatory conditions, such as LPS-induced mortality in mice and sepsis-related mortality in hospitalized patients (12, 13, 45).

Detailed studies at a molecular level are needed to elucidate the functional implications of individual lipid transfer proteins in the biology and pathogenesis of these parasites (46). These results point to the complexity of the interactions between the lipid system, the immune response, and the development of cutaneous leishmaniasis. Understanding these mechanisms could pave the way for innovative therapeutic strategies aimed at modulating the immune response and improving the resolution of the infection.

## Conclusion

Despite the limitations of using animal models for human physiology, the information obtained in this study is unprecedented and represents a step forward in understanding the pathogenesis of CL caused by *Leishmania amazonensis.* The presence of CETP influenced the development of *L. amazonensis* infection in transgenic mice, which may be through the modulation of the immune response. It implied in the decreased parasitism altering the profile of macrophages. This allows us to infer that the progression of the disease to more advanced stages can be contained. These findings shed light on the potential role of the lipid system and lipoproteins in the development of cutaneous leishmaniasis. In this context, the presence of CETP demonstrated a more effective response based on infection control and tissue repair.

## Data availability statement

The data used to support the findings of this study are included within the article. Additional data or information can be requested by contacting the corresponding author.

## Ethics statement

The animal study was approved by National Council for Animal Experimentation Control (CONCEA) and Institutional Animal Care and Use Committee (CEUA) of Faculdade de Medicina da Universidade de São Paulo (FMUSP) protocol number (CEUA- FMUSP #1683/2021). The study was conducted in accordance with the local legislation and institutional requirements.

## Author contributions

FB-D: Investigation, Methodology, Writing – original draft, Writing – review & editing. CO: Investigation, Methodology, Writing – original draft, Writing – review & editing. CS-P: Writing – original draft. BU: Writing – original draft. KS: Writing – original draft. EY-K: Writing – original draft. VN: Writing – review & editing. AD-N: Writing – original draft, Writing – review & editing. WT: Writing – original draft, Writing – review & editing. MS: Writing – original draft, Writing – review & editing. LR: Writing – original draft, Writing – review & editing. HG: Conceptualization, Writing – original draft, Writing – review & editing. PC: Conceptualization, Writing – original draft, Writing – review & editing.
